# Leveraging Artificial Intelligence-Based Applications to Remove Disruptive Factors from Pharmaceutical Care: A Quantitative Study in Eastern Romania

**DOI:** 10.3390/pharmacy14010007

**Published:** 2026-01-09

**Authors:** Ionela Daniela Ferțu, Alina Mihaela Elisei, Mariana Lupoae, Alexandra Burlacu, Claudia Simona Ștefan, Luminița Enache, Andrei Vlad Brădeanu, Loredana Sabina Pascu, Iulia Chiscop, Mădălina Nicoleta Matei, Aurel Nechita, Ancuța Iacob

**Affiliations:** 1 Research Centre in the Medical-Pharmaceutical Field, Department of Pharmaceutical Science, Faculty of Medicine and Pharmacy, “Dunarea de Jos” University of Galati, 800008 Galati, Romania; aelisei@ugal.ro (A.M.E.); mariana.lupoae@ugal.ro (M.L.); alexandra.burlacu@ugal.ro (A.B.); claudia.stefan@ugal.ro (C.S.Ș.); luminitatrufas5@gmail.com (L.E.); 2 Surgical Department, Faculty of Medicine and Pharmacy, “Dunarea de Jos” University of Galati, 800008 Galati, Romania; andrei.bradeanu@ugal.ro; 3 Dental Medicine Department, “Dunarea de Jos” University of Galati, 800008 Galati, Romania; loredana.pascu@ugal.ro (L.S.P.); iulia.chiscop@ugal.ro (I.C.); madalina.matei@ugal.ro (M.N.M.); 4 Clinical Medical Department, Faculty of Medicine and Pharmacy, “Dunarea de Jos” University of Galati, 800008 Galati, Romania; aurel.nechita@ugal.ro

**Keywords:** artificial intelligence, pharmaceutical care, pharmacist, student, barrier, communication

## Abstract

Artificial Intelligence (AI) has increasingly contributed to advancements in pharmaceutical practice, particularly by enhancing the pharmacist–patient relationship and improving medication adherence. This quantitative, descriptive, cross-sectional study investigated Eastern Romanian pharmacists’ perception of AI-based applications as effective optimization tools, correlating it with disruptive communication factors. An anonymous and online questionnaire was distributed to community pharmacists, examining sociodemographic characteristics, awareness of disruptive factors, and the perceived usefulness of AI. The sample included 437 respondents: pharmacists (55.6%), mostly female (83.8%), and aged between 25 and 44 (52.6%). Data analysis involved descriptive statistics and independent *t*-tests. The statistical analysis revealed a significantly positive perception (*p* < 0.001) of AI on pharmacist–patient communication. Respondents viewed AI as a valuable tool for reducing medication errors and optimizing counseling time, though they maintain a strong emphasis on genuine human interaction. Significant correlations were found between disruptive factors—such as noise and high patient volume—and the quality of communication. Participants also expressed an increased interest in applications like automatic prescription scheduling and the use of chatbots. The study concludes that a balanced implementation of AI technologies is necessary, one that runs parallel with the continuous development of pharmacists’ communication skills. Future research should focus on validating AI’s impact on clinical outcomes and establishing clear ethical guidelines regarding the use of patient data.

## 1. Introduction

In the current context of accelerated digitization, pharmaceutical care is redefining its role in the healthcare system, going beyond the traditional function of dispensing medicines [1]. Effective communication between pharmacists and patients is becoming essential not only for therapeutic counseling, but also for preventing medication errors and building trust in the services provided [2]. In both medical and pharmaceutical practice, a number of factors have been reported to disrupt communication [3]. Such factors can be external (the environment: crowding, noise, time; language barriers; also cultural) [4]. Since the COVID-19 pandemic, which represented a significant barrier to the provision of pharmaceutical care, there has been a need to improve the quality of pharmaceutical services for patients, marking the beginning of changes in the pharmacist–patient relationship [5]. The integration of digital technologies, especially artificial intelligence (AI), brings important opportunities, but also ethical and professional challenges [6].

As a field that involves the development of programs and systems capable of performing tasks that mimic human cognitive processes (learning, perception, reasoning, decision-making), AI finds its purpose in the pharmaceutical system because it has a massive database in terms of both human resources and material resources (drug stocks) [7,8].

AI is based on a series of applications that are constantly expanding and influencing almost all areas of activity [9]. In the pharmaceutical field, especially in community pharmacies, these AI applications are aimed in two directions: on the one hand, operational optimization, and on the other hand, improving the communication relationship between pharmacist and patient [10]. This study focuses on the second direction. While a number of applications have already been implemented in highly developed countries, in Romania they are limited [11].

For example, with regard to AI applications such as chatbots and virtual assistants for patients, the UK has implemented the AIVAe (AI Virtual Pharmacy Assistant) application, which provides advice based on the medication administered, thus avoiding adverse effects [12]. In Saudi Arabia, chatbots based on Large Language Models (LLM) are used to quickly generate patient education information about the medications they are taking and the conditions they are suffering [13]. In the US and Asia, there are conversational AI platforms (e.g., Hyro, KeyReply) designed to inform patients about pharmacy hours, remind them to take their medication in order to increase treatment adherence, and quickly upload prescriptions via chat or voice [14]. In Romania, there are only campaigns to inform patients about the promotion of certain medications, but they do not support patients in terms of treatment adherence [15].

AI applications are also being implemented that indirectly improve communication with patients by optimizing workflow, such as the application in the US that automatically generates clinical aspects in the electronic health record (EHR), an application that allows the pharmacist to devote more time to face-to-face counseling sessions, while maintaining eye contact with the patient throughout the communication relationship [16]. Another AI application based on demand prediction algorithms, such as predicting medication needs, ensures that patients receive the medication necessary to treat their condition, thus avoiding frustration and delays in treatment [17].

Another way to improve pharmacist–patient communication used in various countries is the use of a drug interaction detection system based on AI technology [18]. This system works by recording the patient’s history in order to identify potential interactions or allergies, with the pharmacist being informed by an alert and able to quickly initiate a safer and more informed discussion with the patient. In Romania, there are no such applications implemented in the pharmaceutical sector [19].

On the other hand, there are statistical studies in the literature, based on questionnaires, which describe both the perception and the level of awareness of technologies that use AI among groups of pharmacists in the US, students from various pharmacy faculties around the world, such as the Middle East and North Africa, the US, etc., [20,21,22,23].

Therefore, this paper investigates the perception of patients and pharmacists in Eastern Romania regarding the impact of AI on communication within pharmaceutical care. The study analyzes the correlations between communication disruptors, with an emphasis on external factors (noise, time, congestion) and the perceived utility of AI tools as optimization solutions for professional services.

## 2. Methods

### 2.1. Study Design, Setting, and Population

This research was based on the use of a quantitative, descriptive, and cross-sectional design, allowing for a systematic and structured of the opinions of a large sample at a specific point in time, without influencing the variables investigated. The cross-sectional approach aimed to obtain an overview of the level of information, perceptions of usefulness and efficiency, benefits and limitations perceived in pharmacist–patient communication through the use of AI. The descriptive design allowed for the identification and characterization of the socio-demographic features of the respondents, as well as the analysis of how these correlate with the level of openness to digital solutions in pharmacy.

The self-administered questionnaire was the tool used in the study, distributed online (November–December 2025) via the GoogleForms platform, ensuring easy accessibility for participants and the possibility of quickly collecting a large volume of response. All questions included in the survey were marked as mandatory, thus avoiding incomplete responses or missing data.

The questionnaire was validated through a multi-stage process. Specific communication inhibitors (barriers) were selected following a rigorous analysis of the literature on pharmaceutical care. The instrument was then subjected to a content validation procedure by a group of experts, consisting of university pharmacists and practitioners, to confirm that the factors included (e.g., emotional load, noise, time) are representative of the current reality in community pharmacies. Before being applied on a large scale, the questionnaire was pre-tested on a small group of respondents to ensure the clarity of the questions and eliminate ambiguities.

### 2.2. Questionnaire Development

The questionnaire (Appendix A) consisted of 21 clear and concise items, logically organized to facilitate completion and maintain participants’ attention. The questions were closed-ended, with predefined answer options (e.g., using the Lickert Scale), but also with multiple options to capture the nuances of perceptions related to AI applications in pharmacy.

The questionnaire included three sections. The first section contained four socio-demographic items regarding age, gender, provenance (the geographical location of the pharmacy where the respondent carries out their professional activity or internship, in the case of students), and professional status, which were necessary to characterize the group of participants and to analyze the relationships between the respondents’ profiles and the opinions expressed. The inclusion of variables related to digital literacy, language skills, and environmental factors (noise, time, patient volume) enabled a gap analysis of traditional communication barriers. These parameters function as mediating variables, explaining how AI is perceived not only as a technological innovation, but as an adaptive solution designed to neutralize logistical and cognitive disruptors. From simplifying medical terminology and translating protocols in real time to taking over “cold” administrative tasks to free up time for empathetic counseling, these variables underpin the practical usefulness of AI in the specific context of Romanian pharmacies.

The second section of the questionnaire included seven items about recognizing the potential impact of environmental factors on the communication process, as well as recognizing AI-based applications. The last section of the questionnaire (10 items) focused on the level of awareness of the use of AI in pharmaceutical practice, the perception of the usefulness and efficiency of these technologies, the benefits and limitations felt in pharmacist–patient communication, but also the willingness of respondents to accept digital and automated solutions in dispensing medicines and pharmaceutical counseling in order to eliminate disruptive factors. Although seemingly distinct, AI-based purchasing forecasts directly influence communication quality by optimizing operational flow and reducing the pharmacist’s cognitive load. Inventory management automation transforms administrative time into counseling time, eliminating the frustration caused by drug shortages and thus strengthening patient trust. By taking over complex logistical tasks, AI allows pharmacists to move from reactive to proactive and empathetic communication, ensuring treatment availability and personalization of pharmaceutical services.

### 2.3. Participant Recruitment

Participants were recruited online through social networks (Facebook and WhatsApp) by providing the link to connect, as well as from among students at the Faculty of Medicine and Pharmacy in the Eastern region of Romania. To avoid multiple completion and submission of responses, the questionnaire was restricted by requiring the completion of an email address.

### 2.4. Data Analysis

The results were statistically analyzed using SPSS (Statistical Package for the Social Sciences) Statistics, version 26 (IBM Inc., Chicago, IL, USA).

The questionnaire was validated in terms of the internal consistency of the items on the Lickert Scale by calculating the Cronbach coefficient, which yielded a value of 0.914. Descriptive tests were also applied to test the hypotheses, calculating the frequency (N), percentage (%), mean (M), and standard deviation (SD), as well as the *t*-test. The Kolmogorov–Smirnov test was used to analyze the normality.

## 3. Results

### 3.1. Demographic Characteristics

The group of participants consisted of 437 respondents who completed a structured questionnaire, reflecting a predominantly young profile, with 52.6% of respondents under the age of 45. This age group is essential in the implementation of pharmaceutical communication. It is important to adapt messages and campaigns to meet the needs of this segment, but without neglecting men, in order to reduce information inequalities and promote inclusive services in pharmacies. The predominance of female respondents (approx. 90%) is not a sampling bias but a reflection of the actual professional demographics in Romania, where the pharmaceutical workforce is overwhelmingly female.

The majority come from urban areas (79.2%) suggesting a higher degree of exposure to technology and modern medical services. This urban preponderance highlights greater access to pharmacies and medical services in cities, but also a potential need for strategies dedicated to rural areas. Pharmaceutical communication must include channels that are accessible and relevant to the rural population, thus reducing differences in information and access to quality counseling. We acknowledge the lower response rate from rural areas, which may be attributed to differences in digital infrastructure and workload distribution.

Additional cohort analyses, respectively, independent samples *t*-test, were performed to assess the influence of gender and place of residence on the perception of AI. The results did not indicate any statistically significant differences between men and women (*p* = 0.51), neither between respondents from rural and urban areas (*p* = 0.32). Ethics and professional vision in pharmacy are standardized, regardless of gender, and communication challenges (lack of time, noise, patient volume) are universally felt by pharmacists, regardless of the geographical location of the pharmacy. These data confirm that, although the sample shows an uneven distribution of these variables, the view of the usefulness of AI in pharmaceutical care is uniform across the entire study group. The composition of the sample included mostly students and specialists in the pharmaceutical field, indicating a high level of familiarity with specialized terminology. This research focused exclusively on licensed pharmacists, excluding pharmacy technicians/assistants. This choice was based on the fact that pharmacists bear the ultimate clinical and legal responsibility for adopting AI tools in patient care. Nevertheless, we recognize that including technicians in future studies would provide a more holistic view of the operational impact of AI on the entire pharmacy team.

Regarding the professional status of respondents, there were no statistically significant differences between students’ perceptions and those of pharmacists regarding the impact of AI on communication. To validate the use of the consolidated sample, a comparative analysis was performed between the subgroups of students and pharmacists. Independent samples *t*-test revealed a high homogeneity of responses (*p* = 0.42), confirming that professional status did not significantly influence perceptions of artificial intelligence. This absence of variance allowed the sample to be treated as a single group in subsequent analyses. This homogeneity suggests that the current academic training of students aligns with the professional perspective of practicing pharmacists regarding technological integration.

This demographic structure is relevant to the research objectives, as it allows for the capture of perceptions of a segment of the population with high potential for adaptation and increased use of innovative technologies and AI-based applications in pharmaceutical practice. The profile of the participants, illustrated in Table 1, also suggests a greater willingness to change, innovate, and integrate digital solutions into pharmaceutical services, while highlighting the importance of adapting AI implementation strategies to the specific characteristics of the target audience.

### 3.2. Recognition of Disruptive Factors Involved in the Communication Process During Pharmaceutical Care

**Figure 1:**
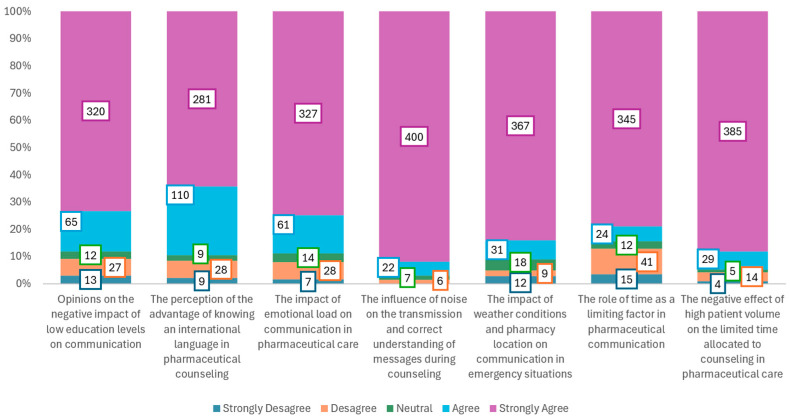
Perception of the involvement of disruptive factors in the pharmacist–patient communication relationship.

Figure 1 shows the participants responses regarding the recognition of external (linguistic, emotional, noise, crowding, time) barriers on a 5-point Lickert Scale.

The question regarding the recognition of the low level of education of the patient, the pharmacist, or both parties as a disruptive factor in the communication process in the provision of pharmaceutical care obtained a majority who stated that they strongly agree (73.2%), agree (14.8%), neutral (2.7%), disagree (6.1%), and strongly disagree (2.9%). This distribution highlights the importance of education in pharmaceutical communication. Professionals in the field need to develop continuing education programs for staff and educational campaigns for patients. The lack of clear and accessible language can lead to confusion, reducing adherence to treatment. Interdisciplinary collaboration cand reduce barriers, leading to empathetic and effective counseling tailored to each person’s level of understanding.

When asked about the advantage of knowing an international language in pharmaceutical practice, most respondents strongly agreed (64.3%), followed by 25.7% who agree, 2.0% who are neutral, 6.4% who disagree, and 2.0% who strongly disagree. The results suggest a broad consensus on the importance of language skills in pharmaceutical counseling. Providing services in multiple languages contributes to inclusion and equal access. Pharmacists can thus clearly explain treatments to foreign or minority patients. Multilingual training is becoming a necessity in a globalized context, reducing the risk of communication errors and increasing patient satisfaction.

On the other hand, the emotional burden was also considered by the majority (74.8%) to be a factor that can positively or negatively influence the communication relationship in the pharmaceutical act. The data reflects a clear awareness of the effect of emotions on the quality of interaction. Patients’ emotions can amplify stress or mistrust, requiring emphatic and personalized approaches. Pharmacists need to develop socio-emotional skills, listen actively, and offer support without judgment.

Major obstacles to patient communication, such as noise, weather conditions and the location of the pharmacy, time and congestion, were reported by the majority of respondents, with 91.5%, 83.9%, 78.9% and 88.1%, respectively, agreeing that they have a negative impact on pharmaceutical counseling.

Noise and interruptions caused by street sounds or communication devices compromise the quality of communication and increase the risk of errors. Combined with possible hearing problems in patients, this can result in the information provided by the pharmacist not being received correctly. Extreme weather conditions, such as blizzards, floods, or very low temperatures, together with the unfavorable geographical location of some pharmacies, especially in rural areas, can significantly affect immediate communication in emergency situations. The digitization of pharmaceuticals services through AI, using mobile applications, notifications, or telepharmacy services, can save lives in critical situations. It is necessary to expand online counseling services and home deliveries. This can overcome barriers caused by distance or unfavorable weather, ensuring continuity of care and patient safety.

Based on the hypothesis that environmental conditions such as noise and location, along with the large volume of patients accessing pharmaceuticals services, are recognized as obstacles to pharmacist–patient communication, Table 2 provides a detailed analysis of respondents’ perceptions of the predominant barriers in the pharmacist–patient communication process.

The average responses indicated that participants generally agreed with the statements related to barriers in pharmacist–patient communication. For the item on noise, the average of 3.55 (on a scale of 1 to 4) suggests strong agreement that noise interferes with the transmission and correct understanding of messages in pharmacies (Table 2). Weather conditions or the location of the pharmacy were recognized as significant obstacles, but somewhat more moderately, with an average of 3.17, still indicating a clear perception that they limit emergency communication. High patient volume scored an average of 3.49, showing a high level of agreement that crowding reduces the time available for effective counseling.

These average scores above 3 confirm the hypothesis that environmental conditions such as noise and location, along with high patient volume, are recognized as significant barriers to pharmacist–patient communication. The results support the need for strategies to reduce these barriers in order to improve the quality of counseling and patient safety in the medication dispensing process.

For the item “Does noise the correct transmission and understanding of messages during counseling in pharmacies?”, the results show a *t*-value is 101.846, significance *p* < 0.001, with an estimated mean of 3.554 (95% confidence interval: 3.49–3.62). This indicates a very clear agreement among respondents with the statement, well above the neutral threshold, demonstrating that noise is perceived as a major obstacle to communication (Table 3).

For the question “Do weather conditions and/or the location of the pharmacy prevent immediate communication in emergency situations?”, the *t*-value is 69.912, with *p* < 0.001, and the mean response is 3.172 (95% confidence interval: 3.08–3.26). Respondents tend to agree here as well, although the level of agreement is somewhat more moderate, indicating that location and weather conditions are recognized as real barriers to communication.

For the item “Does the high volume of patients contribute to limiting the time allocated for pharmaceutical counseling?”, the *t*-value is 84.888, with *p* < 0.001 and a mean of 3.485 (95% confidence interval: 3.40–3.57). The results highlight a strong agreement among participants that crowding reduces the quality and time devoted to pharmacist–patient counseling.

### 3.3. Assessment of Perceptions of AI Applications and Their Associated Benefits

Continuing education for pharmacy staff and clear clarification of the role and limitations of AI-based applications are recommended to ensure effective and responsible adoption of new technologies. AI streamlines routine operations, allowing the pharmacist to tailor communication and address individual patient needs. Table 4 shows respondents’ opinions on the usefulness of certain types of AI applications. 123 respondents opted for the implementation of programs for information and automatic analysis of drug interactions and adverse reactions, followed by applications that provide for the personalization and automatic design of a drug treatment plan, with a percentage of 18.5%. Only 5 respondents considered automatic prescription programming.

**Figure 2:**
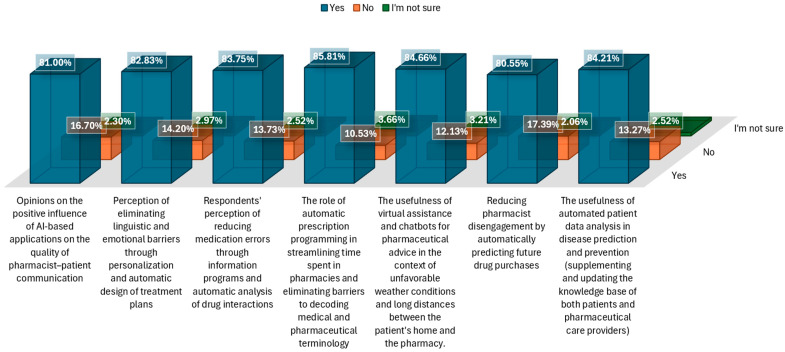
Perception of the influence of AI-based applications in eliminating disruptive factors in communication.

More than half of respondents (81.0%) believe that AI-based applications could positively influence the quality of communication between pharmacists and patients, while 2.2% are unsure and 16.7% say no (Figure 2). A clear majority recognize the positive potential of AI-based applications in communication quality. However, a quarter of respondents indicate uncertainty, suggesting a need for education and clarification of the concrete benefits of AI technologies in the patient relationship.

More than half of participants (82.8%) believe that personalization and automatic design of a medication treatment plan could eliminate language and emotional barriers, 2.9% are unsure, and 14.1% say no. The majority of respondents believe that AI can reduce language and emotional barriers. This perception supports the development of patient-centered solutions that are adaptable to linguistic and cultural diversity. Most respondents (83.7%) believe that medication errors can be eliminated through automated programs that analyze drug interactions, 2.5% are unsure, and 13.7% say no. Most see a clear role for AI in increasing therapeutic safety by reducing human error. This validates investments in automatic alert systems and complex analysis of drug interactions.

Regarding the question, “Does automatic prescription programming help reduce the time spent in the pharmacy and eliminate barriers to decoding medical and pharmaceutical terminology?”, the majority of participants (85.8%) believe that automatic prescription scheduling reduces the time spent in the pharmacy and eliminates barriers to decoding medical terminology, 3.6% are unsure, and 10.5% say no. This is the highest consensus in the entire set of questions. Respondents strongly appreciate the practical value of automation for efficiency and clarity in the pharmaceutical process.

More than half of respondents (84.6%) believe that virtual assistance and chatbots are useful in adverse weather conditions or over long distances, 3.2% are unsure, and 12.1% say no. Our results indicate a generally positive perception of the usefulness of AI in facilitating access to care. This view, uniformly supported by both urban and rural respondents (*p* > 0.05), underpins the need for teleconsultation solutions to overcome physical accessibility barriers specific to isolated areas or adverse weather conditions.

Regarding the question “Can the pharmacist’s lack of interest in the patient be eliminated by automatically predicting future drug purchases?”, three-quarters of participants (80.5%) believe that the pharmacist’s lack of interest can be eliminated by automatically predicting future drug purchases, 2.0% are unsure, and 17.3% answered no. More than half believe in the potential of AI to anticipate patient needs and personalize interactions, but a third are skeptical or unsure, highlighting the need to clarify the ethics and confidentiality of these tools.

Most respondents (84.2%) believe that disease predictability and prevention through automated data analysis helps to update the knowledge of patients and pharmaceutical staff, 2.5% are unsure, and 13.2% say no. The results confirm the interest in using data analysis for educational purposes, both for patients and pharmacists. They support the idea of a digital pharmacy as a hub for personalized information.

According to Table 5, most respondents (33.2%) consider that the main advantage of artificial intelligence applications in the pharmacist–patient relationship is quick access to information, followed by the elimination of human error (18.1%), cost reduction (16.0%), unconditional availability (15.3%), and adaptability to all patient types (12.8%). Rapid access to information is perceived as the key factor in improving dialog with patients, suggesting the need for AI tools that support quick and accurate decisions. This prioritization highlights the expectation for informed, transparent, and personalized pharmaceutical care.

The hypothesis that “The use of artificial intelligence in pharmaceutical activities will significantly improve pharmacist–patient communication by reducing consultation time, increasing the quality of information provided, and strengthening patient confidence in pharmaceutical services, provided that human interaction is maintained” is confirmed by the results of the descriptive statistics highlighted in Table 6.

The average values obtained show a high level of agreement among respondents regarding the role of artificial intelligence in improving pharmacist–patient communication, with an average of 1.48 indicating a clearly positive perception of AI’s potential to increase the quality of dialog (Table 6). Regarding automatic prescription scheduling, the average of 1.58 suggests that participants recognize the clear benefits in reducing the time spent in the pharmacy and simplifying specialized language. Similarly, the average of 1.76 reflects fairly solid acceptance of the idea that automatic data analysis can update the knowledge of patients and pharmacy staff, supporting overall openness to the integration of AI into pharmacy services.

Following the application of the *t*-test, the results obtained were included in Table 7. Thus, for the question “Could artificial intelligence positively influence the quality of communication in the pharmacist–patient relationship?”, a very high *t*-value was obtained (*t* = 45.802, *p* = 0.000) with an average difference of 1.481 and a confidence interval between 1.42 and 1.54. This shows that respondents clearly evaluate this statement positively in relation to the zero-reference value, suggesting strong agreement with the beneficial role of AI in improving communication. In the case of the question “Does automatic prescription scheduling help reduce the time spent in the pharmacy and eliminate barriers to decoding medical and pharmaceutical terminology?”, the result is *t* = 47.141 with *p* = 0.000 and an average difference of 1.581 (range 1.52–1.65). The result highlights a significant agreement among respondents regarding the impact of AI in reducing consultation time and facilitating understanding of terminology. For the item “Does the predictability of diseases and their prevention through automatic analysis of patient data complement and update the knowledge base of both patients and pharmaceutical care staff?”, the test indicates *t* = 45.821, *p* = 0.000, with an average difference of 1.757 and an interval of 1.68–1.83. This underscores strong support for the idea that AI contributes to increasing the quality and clarity of information provided to patients.

Analyzing these results, all *t*-values are very high and *p* < 0.001 in all cases, demonstrating that the differences from zero are extremely statistically significant. The positive means and narrow confidence intervals, all clearly above zero, confirm that respondents perceive the use of artificial intelligence in pharmacy as having a substantial beneficial impact on the quality of communication, reducing consultation time, and strengthening patient confidence in pharmaceutical services.

While the current implementation of artificial intelligence in pharmacies in eastern Romania is still in its early stages, the study reveals a remarkable professional openness to this technology. Respondents identified automatic prescription scheduling and predictive analysis of patient data as the main functions capable of optimizing pharmaceutical care. This positive perception (*p* < 0.001) suggests that pharmacists do not view AI as a substitute for human interaction, but rather as an essential tool for managing pragmatic barriers such as excessive workload and time constraints.

Thus, the results obtained support the research hypothesis from a professional perception perspective, indicating that pharmacists and students foresee an improvement in communication with patients through the integration of AI. These data reflect a high degree of confidence in the technology’s potential to optimize consultation time and information quality. However, definitive confirmation of these benefits requires future prospective studies that objectively measure the performance of AI platforms in the clinical setting, using samples that are balanced in terms of demographics and professional experience.

## 4. Discussion

Pharmacies and healthcare systems in general are undergoing a period of transition in which the digitization of processes is becoming a necessity, not just a competitive advantage [24]. The results obtained in this study highlight a clear trend among pharmaceutical professionals toward embracing artificial intelligence as a tool to support their daily work [25,26].

The comparative analysis between different age groups did not reveal any statistically significant differences in terms of reluctance towards AI (*p* > 0.05). This result suggests that openness to technology integration is a cross-cutting attitude, shared equally by younger generations and experienced pharmacists, thus invalidating the hypothesis of a generational barrier to the adoption of digital innovations in the context studied [27]. Most respondents perceive artificial intelligence (AI) as a useful tool for improving pharmacist–patient communication, with an emphasis on reducing counseling time and eliminating human error. However, this positive perception is accompanied by reservations about completely replacing human interaction, which confirms the initial hypothesis regarding the need for a balance between technology and the human factor. The literature emphasizes that AI-based technologies have the ability to support decision-making processes, identify dangerous drug interactions, and suggest safer alternatives, thus contributing to patient safety [28].

Thus, the distribution of preferences regarding AI applications shows greater interest in drug interaction analysis and treatment personalization programs rather than logistics management applications. This trend suggests that participants are more concerned with the direct impact on health and safety than with streamlining administrative processes. With regard to medication errors, most respondents recognized the value of programs that provide information and automatically analyze drug interactions in preventing these risks. Similarly, the idea that personalizing medication treatment plans can eliminate language and emotional barriers reflects a central goal of modern medicine and pharmacy: tailoring services to individual patient needs. AI enables the integration of relevant patient data and the generation of personalized recommendations, thereby increasing the chances of treatment adherence and reducing the risk of abandonment or misunderstanding [29].

The predictability of diseases and their prevention through automatic analysis of patient data is another area of interest confirmed by respondents. This functionality supports the transition from a reactive to a proactive approach to public health, enabling early risk identification, personalization of preventive interventions, and long-term cost reduction [30].

Respondents’ preference for applications such as inventory management automation, automatic collection and analysis of medical data, personalization of treatment plans, virtual assistance, and chatbots shows a concern for streamlining administrative activities, reducing errors, and improving patient services [31]. The literature outlines the same direction of transformation, in which repetitive and time-consuming tasks are taken over by intelligent systems, freeing up human resources for activities with higher added value [32].

At the same time, the study results confirm the idea that patients and healthcare professionals value rapid access to information and reducing the risk of error [33]. Beyond the obvious benefits of lower operating costs, eliminating human error is seen as a key part of quality pharmaceutical services.

The unconditional availability of services, another advantage identified by respondents, aligns perfectly with the trend of increasing patient expectations regarding the accessibility of healthcare services. The AI-assisted digital pharmacy responds to this need by extending services beyond traditional time and space constraints. In adverse weather conditions or when the distance to the pharmacy is long, virtual assistance and chatbots become valuable tools that maintain continuity of care and support the inclusion of patients who might otherwise be marginalized by logistical constraints [34].

Furthermore, the results regarding automatic prescription scheduling, accepted by most respondents as a means of reducing the time spent in the pharmacy and clarifying medical and pharmaceutical terminology, confirm the need for solutions that simplify the patient’s journey through the healthcare system. The literature suggests that automating these processes reduces pressure on pharmacists, prevents confusion caused by illegible handwriting or specialized medical terminology, and supports a smoother and more satisfying experience for the patient [35]. Pharmacies and healthcare systems in general are in a period of transition in which the digitization of processes is becoming a necessity, not just a competitive advantage [36]. Interpreting the data, it can be seen that noise, high patient volume, and limited time are identified as the most important disruptive factors, with significant percentages above 75%. This suggests that the pressure on the pharmacist and patient in the current environment is real and constant, and technological solutions are perceived as a possible way to optimize. This finding is in line with the conclusions of other authors, who have emphasized the role of AI in reducing repetitive tasks [37].

Overall, the data collected through this questionnaire aligns with general trends toward digitization and personalization of pharmaceutical services, confirming that the target audience has a growing interest in the use of advanced technologies that can improve the safety, efficiency, and quality of the pharmacist–patient relationship [38]. However, the literature suggests that the success of these innovations depends not only on technological availability, but also on the level of education and trust of users, the adaptation of infrastructure, and ethical and legal regulations to ensure data protection and equitable access [39]. The effective implementation of artificial intelligence in pharmacies in eastern Romania thus requires a concerted effort, in which both the education system and the community pharmacy infrastructure support digital transformation in a sustainable and inclusive manner.

Consequently, by investigating the intersection of digital innovation and pharmaceutical practice, this paper demonstrates how AI-driven tools can bypass traditional communication barriers in Romanian pharmacies. The novelty of this work lies in its empirical evidence that environmental challenges actually drive the demand for automated technologies, positioning AI as a strategic asset for enhancing the clinical counseling experience.

This study also presents several methodological limitations that must be taken into account. First, selection bias was influenced by the use of social media platforms for recruitment, which led to a disproportionate representation of women and urban respondents. Although this profile partially reflects the demographics of the profession in Romania, it limits the generalization of the results at the national level. Second, the geographical delimitation to the eastern region of Romania may introduce specific variations in digital infrastructure. Another important limitation is the consolidation of data from students and practicing pharmacists; although statistical tests did not indicate significant differences (*p* = 0.42), we recognize that the limited experience of students may nuance the perception of pragmatic barriers. Furthermore, the study focused on community pharmacies without segmenting the responses of hospital pharmacists, which could offer a different perspective on technological needs. The study focused on community pharmacies because that is where interaction with patients is most intense and the communication barriers identified (noise, time) are most significant.

## 5. Conclusions

Effective communication between pharmacists and patients is essential for providing quality pharmaceutical services, contributing to the safety and success of drug treatments. In the context of digitalization and the emergence of AI-based technologies, pharmaceutical care is undergoing a profound transformation, requiring a balance between innovation and maintaining direct human relationships. AI does not automate the pharmacist’s tasks, but instead allows more time to be allocated to personalized and empathetic counseling for each patient, strengthening the relationship and trust with them. Repetitive requests related to dosage, contraindications, or drug interactions are reduced, as AI can provide instant answers in the form of warnings on smart devices, improving treatment adherence. These systems also raise significant privacy concerns. The risk of a security breach can be severe and compromising for sensitive patient data. Currently, the most popular chatbots do not comply with health standards such as the Health Insurance Portability and Accountability Act (HIPAA) in the United States or data protection regulations in Europe (GDPR). Pharmacists must be trained to monitor AI outputs, assess their limitations, and intervene appropriately. Regular audits and feedback loops are essential to maintaining reliability. This paper highlights the importance of responsible integration of AI in pharmacies in Eastern Romania, with an emphasis on maintaining personalized, clear, and empathetic communication. The implementation of these technologies must be accompanied by clear regulations, adequate professional training, and constant attention to data protection and professional ethics to ensure a modern, accessible, and safe pharmaceutical system for all patients.

## Figures and Tables

**Table 1:** Respondent profile.

Variables	Categories	Frequency (N)	Percent (%)	Valid Percent (%)	Cumulative Percent (%)
Age (years)	18–24	96	22.0	22.0	22.0
	25–44	230	52.6	52.6	74.6
	45–65	101	23.1	23.1	97.7
	≥65	10	2.3	2.3	100
Gender	Female	366	83.8	83.8	83.8
	Male	71	16.2	16.2	100
	Other	0	0	0	100
Provenance	Urban	346	79.2	79.2	79.2
	Rural	91	20.8	20.8	100
Professional status	Pharmacist	243	55.6	55.6	55.6
	Student	194	44.4	44.4	100

**Table 2:** Descriptive statistics for perceptions of barriers to pharmacist–patient communication.

One-Sample Statistics	N	M	SD	Std. Error Mean
Noise hinders the transmission and correct understanding of messages during counseling in pharmacies.	437	3.55	0.729	0.035
Weather conditions and/or the location of the pharmacy prevent immediate communication in emergency situations.	437	3.17	0.948	0.045
The large volume of patients contributes to limiting the time allocated for pharmaceutical counseling.	437	3.49	0.858	0.041

**Table 3:** Results of the *t*-test on perceptions of barriers to pharmacist–patient communication.

One-Sample Test	Test Value = 0					
	*t*	df	Sig. (2-Tailed)	Difference Between Means	95% Confidence Interval of the Difference	
					Low	High
Noise hinders the transmission and correct understanding of messages during counseling in pharmacies	101.846	436	0.000	3.554	3.49	3.62
Weather conditions and/or the location of the pharmacy prevent immediate communication in emergency situations	69.912	436	0.000	3.172	3.08	3.26
The large volume of patients contributes to limiting the time allocated for pharmaceutical counseling.	84.888	436	0.000	3.485	3.40	3.57

**Table 4:** Artificial intelligence applications that are effective and useful by respondents.

Number of Participants (N = 437)	Categories	N (%)
Which of the following applications of artificial intelligence do you consider useful and effective in pharmacies?	Personalization and automatic design of a medication treatment plan	81 (18.5%)
	Programs for automatic information and analysis of drug interactions and adverse reactions	123 (28.1%)
	Automation of drug inventory management	9 (2.1%)
	Automatic prescription scheduling	5 (1.1%)
	Virtual assistance and chatbots for pharmaceutical advice	11 (2.5%)
	Automatic preparation of magistral and officinal preparations	8 (1.8%)
	Automatic collection and analysis of data in patients’ medical history and complaints about pharmaceutical assistance services	23 (5.3%)
	Automatic prediction of future drug purchases	60 (13.7%)
	Automatic preparation, packaging, and dispensing of drugs	9 (2.1%)
	Predictability of diseases and their prevention through data analysis	80 (18.3%)
	None	5 (1.1%)
	All	23 (5.3%)

**Table 5:** Perceived benefits of using AI applications in pharmacist–patient communication.

Categories	Frequency (N)	Percent (%)	Valid Percent (%)	Cumulative Percent (%)
24/7 support	15	3.4	3.4	3.4
Quick access to information	145	33.2	33.2	36.6
Adaptability to all patient types	56	12.8	12.8	49.4
Reduction in communication errors	1	0.2	0.2	49.7
Lower costs	70	16.0	16.0	65.7
Unconditional availability	67	15.3	15.3	81.0
Accessibility and inclusion	4	0.9	0.9	81.9
Elimination of human error	79	18.1	18.1	100.0
Total	437	100.0	100.0	

**Table 6:** Descriptive statistics for perceptions of the benefits of AI in pharmacist–patient communication.

One-Sample Statistics	N	Mean	Std. Deviation	Std. Error Mean
Artificial intelligence could positively influence the quality of communication in the pharmacist–patient relationship.	437	1.48	0.676	0.032
Automatic prescription scheduling helps reduce the time spent in the pharmacy and eliminates some barriers to decoding medical and pharmaceutical terminology.	437	1.58	0.701	0.034
Predicting and preventing diseases through automatic analysis of patient data complements and updates the knowledge base of both patients and pharmaceutical care staff.	437	1.76	0.802	0.038

**Table 7:** *t*-test results for assessing perceptions of the impact of AI on pharmacist–patient communication.

One-Sample Test	Test Value = 0					
	t	df	Sig. (2-Tailed)	Difference Between Means	95% Confidence Interval of the Difference	
					Low	High
Artificial intelligence could positively influence the quality of communication in the pharmacist–patient relationship	45.802	436	0.000	1.481	1.42	1.54
Automatic prescription scheduling helps reduce the time spent in the pharmacy and eliminates barriers to decoding medical and pharmaceutical terminology	47.141	436	0.000	1.581	1.52	1.65
Predicting and preventing diseases through automatic analysis of patient data complements and updates the knowledge base of both patients and pharmaceutical care providers.	45.821	436	0.000	1.757	1.68	1.83

## Data Availability

The original contributions presented in this study are included in the article. Further inquiries can be directed to the corresponding authors.

## Appendix A

### Questionnaire on the Influence of AI-Based Applications on Disruptive Factors Involved in Pharmaceutical Care

Section 1: Socio-demographic information

1. Age:
  ○ 18–24 years
  ○ 25–44 years
  ○ 45–65 years
  ○ After 65 years
2. Gender:
  ○ Male
  ○ Female
  ○ Other
3. Residence:
  ○ Urban
  ○ Rural
4. Professional status:
  ○ Student
  ○ Pharmacist

Section 2: Recognition of disruptive factors involved in the communication process during pharmaceutical care

**Table A1:** Please indicate your level of agreement with the following statements regarding the impact of disruptive factors in the communication process during pharmaceutical care (1 = Total disagreement, 5 = Total agreement).

No.	Items	Strongly Disagree	Disagree	Neutral	Agree	Strongly Agree
5	A low level of education of the patient or pharmacist/pharmacy assistant/cosmetician or both parties can lead to poor and ineffective communication.	⎕	⎕	⎕	⎕	⎕
6	Knowledge of an international language in pharmaceutical practice is an advantage for advising a wide range of patients on pharmaceutical care.	⎕	⎕	⎕	⎕	⎕
7	The course of communication in pharmaceutical assistance can be positively or negatively influenced by the emotional charge of the participants in the dialog.	⎕	⎕	⎕	⎕	⎕
8	Noise hinders the transmission and correct understanding of messages during counseling in pharmacies.	⎕	⎕	⎕	⎕	⎕
9	Weather conditions and/or the location of the pharmacy prevent immediate communication in emergency situations.	⎕	⎕	⎕	⎕	⎕
10	Time is a limiting factor in effective communication in pharmaceutical care.	⎕	⎕	⎕	⎕	⎕
11	The large volume of patients contributes to limiting the time allocated for counseling in pharmaceutical care.	⎕	⎕	⎕	⎕	⎕

Section 3: Recognition of artificial intelligence-based applications and their influence on communication disruptors

12. Have you heard about the use of artificial intelligence in pharmaceutical assistance?

○ Yes
○ No
○ I’m Not Sure

13. Which of the following applications of artificial intelligence do you consider useful and effective in pharmacies?

○ Customization and automatic design of a medication treatment plan
○ Programs for information and automatic analysis of drug interactions and adverse reactions
○ Automation of medicine stock management
○ Automatic recipe programming
○ Virtual assistance and chatbots for pharmaceutical consulting
○ Automatic manufacture of extemporaneous and officinal preparations
○ Automatic collection and analysis of data on patients’ medical history and complaints about pharmaceutical services
○ Automatic prediction of future drug purchases
○ Automatic preparation, packaging, and dispensing of medications
○ Predicting and preventing diseases through data analysis
○ None
○ All

**Table A2:** Please select your opinion on the influence of AI-based applications on communication disruptors.

No.	Items	Yes	No	I’m Not Sure
14	Could applications based on artificial intelligence positively influence the quality of communication in the pharmacist–patient relationship?	⎕	⎕	⎕
15	Could the personalization and automated design of a medication treatment plan eliminate language barriers and emotional barriers?	⎕	⎕	⎕
16	Can medication errors be eliminated by using programs that provide information and automatically analyze drug interactions?	⎕	⎕	⎕
17	Does automatic prescription scheduling help reduce the time spent in the pharmacy and eliminate barriers to understanding medical and pharmaceutical terminology?	⎕	⎕	⎕
18	Could virtual assistance and chatbots for pharmaceutical advice be beneficial in adverse weather conditions or when there is a long distance between the patient’s home and the pharmacy?	⎕	⎕	⎕
19	Can the pharmacist’s lack of interest in the patient be eliminated by automatically predicting future drug purchases?	⎕	⎕	⎕
20	Does the predictability of diseases and their prevention through automatic analysis of patient data complement and update the knowledge base of both patients and pharmaceutical care providers?	⎕	⎕	⎕

21. Which of the benefits of using artificial intelligence applications do you consider to be the greatest advantage in the pharmacist–patient communication relationship?

○ Lower costs
○ Quick access to information
○ Unconditional availability
○ Elimination of human error
○ Adaptability to all types of patients
